# Sandhoff Disease Associated With Bipolar Disorder: A Case Report From the United Arab Emirates

**DOI:** 10.7759/cureus.115548

**Published:** 2026-08-31

**Authors:** Ansam Abdelrahman, Maryam Alowais, Ahmed Elamin, Aaya E Ahmed, Adiba Rakhange, Ahmed Darweesh, Abdul Jabbar Adi, Ahmad Otour

**Affiliations:** 1 Psychiatry and Behavioral Sciences, Graduate Medical Education, Mohammed Bin Rashid University of Medicine and Health Sciences, Dubai Health, Dubai, ARE; 2 Medicine, Graduate Medical Education, Mohammed Bin Rashid University of Medicine and Health Sciences, Dubai Health, Dubai, ARE; 3 Medicine, Dubai Medical University, Dubai, ARE; 4 Medicine, Dubai Hospital, Dubai Health, Dubai, ARE; 5 Clinical Sciences, University of Sharjah, Sharjah, ARE

**Keywords:** adult-onset neurodegeneration, bipolar disorders, gm2 gangliosidosis, hexb mutation, neuropsychiatric manifestation, sandhoff disease

## Abstract

Late‑onset Sandhoff disease is a rare lysosomal storage disorder that may present with neuropsychiatric features. This report describes a 38-year-old woman with an established diagnosis of bipolar I disorder who developed progressive neurological symptoms alongside an acute manic and psychotic episode. Her presentation included motor incoordination, speech difficulties, weakness, and functional decline, accompanied by cerebellar signs on neurological examination and structural changes on neuroimaging. Genetic evaluation confirmed a pathogenic variant consistent with Sandhoff disease. The acute psychiatric episode was managed with antipsychotic and mood‑stabilizing treatment, leading to resolution of manic symptoms and subsequent psychiatric stability during rehabilitation and follow‑up. This case highlights the importance of considering an underlying metabolic or neurogenetic disorder when psychiatric symptoms occur in the context of progressive neurological findings, as such comorbidity can complicate diagnosis and management and requires a multidisciplinary approach.

## Introduction

Sandhoff disease is a rare lysosomal storage disorder and a subtype of GM2 gangliosidosis caused by pathogenic variants in the HEXB gene on chromosome 5q13. The HEXB gene encodes the beta subunit shared by beta-hexosaminidase A and beta-hexosaminidase B, enzymes involved in the degradation of GM2 gangliosides and other glycosphingolipids. Pathogenic variants result in impaired degradation and intracellular accumulation of GM2 gangliosides, leading to progressive neuronal dysfunction and neurodegeneration [1,2].

The clinical presentation of Sandhoff disease varies according to age of onset. Infantile disease typically presents during early childhood with developmental regression, hypotonia, motor impairment, exaggerated startle responses, and progressive neurological deterioration. Juvenile and adult-onset forms may have a more slowly progressive course and may be characterized by ataxia, weakness, dysarthria, cognitive impairment, and psychiatric manifestations [3-5].

Adult-onset GM2 gangliosidosis can be particularly difficult to diagnose because neurological symptoms may initially be attributed to more common neurological or psychiatric conditions. Neuropsychiatric manifestations described in late-onset GM2 gangliosidosis include mood disturbance, psychosis, behavioral changes, delusions, ideas of reference, and hallucinations [4,6]. Psychiatric symptoms may therefore precede or coexist with progressive neurological manifestations.

Bipolar disorder is a major psychiatric disorder characterized by recurrent episodes of mania, hypomania, and/or depression [7]. The coexistence of a primary psychiatric disorder with a progressive neurodegenerative or metabolic disease may complicate diagnosis and management, as psychiatric symptoms can stem from more than one underlying process.

We present a 38-year-old woman with an established diagnosis of bipolar I disorder who subsequently developed progressive neurological impairment and was diagnosed with genetically confirmed Sandhoff disease. To our knowledge, this represents the first reported case of Sandhoff disease with co-occurring bipolar disorder documented in the United Arab Emirates.

## Case presentation

We present the case of a 38-year-old woman of Iranian nationality, resident in the United Arab Emirates, diagnosed with bipolar I disorder in 2008. She presented to the emergency department with a five-day history of significant manic symptoms, progressing to agitation and the need for hospitalization. Symptoms included abnormally increased energy, expansive and irritable mood, decreased need for sleep, and talkativeness with a flight of ideas. She also exhibited impulsive spending through online shopping, psychomotor agitation, persecutory delusions toward her family, and reported possible auditory hallucinations. The patient had a history of self-harm but denied any suicidal thoughts or substance abuse. Her medical history included a diagnosis of ataxia in 2019 and a recent diagnosis of Sandhoff disease confirmed by genetic testing performed at a commercial genetics laboratory in the United Arab Emirates.

Whole-exome sequencing (WES) was performed with blood samples from the proband and her siblings. WES revealed a homozygous mutation in the HEXB gene on chromosome 5 (c.1514G>A pathogenic variant, encoding p.Arg505Gln) in the patient, while the rest of her siblings were heterozygous carriers of the HEXB mutation. The patient's HEXB variant was a homozygous pathogenic missense variant associated with infantile, juvenile, and adult forms of Sandhoff disease (Table 1).

**Table 1:** Primary finding * Genomic position based on the Genome Reference Consortium Human Genome Build 37 (GRCh37). ** SIFT and PolyPhen-2 are computational tools that predict whether a genetic variant is likely to be damaging to protein function; both predicted this variant to be damaging. *** The frequency of this variant in the general population, based on the gnomAD population database; a frequency this low is consistent with a rare, disease-causing variant. **** This variant had not been previously recorded in the internal reference database used for comparison. ***** Variant pathogenicity was classified according to ACMG guidelines, the standard framework used to grade the likelihood that a genetic variant causes disease. Hom: homozygous, Het: heterozygous, OMIM: Online Mendelian Inheritance in Man, ACMG: American College of Medical Genetics and Genomics

Gene (genomic position*)	DNA change, protein change (transcript)	Zygosity	dbSNP ID	In silico predictions**	Allele frequency***	FirstVar****	Disorder (OMIM#, inheritance)	Variant classification*****
HEXB (Chr5:74016473)	c.1514G>A, p.Arg505Gln (NM_000521.4)	Hom	rs121907983	Damaging (SIFT and PolyPhen-2)	0.003656%, 0 Hom 4 Het	Not previously reported	Sandhoff disease: infantile, juvenile, and adult forms (OMIM 268800, autosomal recessive)	Pathogenic

The patient is the child of first-degree consanguineous parents and has a strong history of consanguinity within each parent's family tree. She was born full term via normal delivery and is the second of four siblings, none of whom had a significant medical or psychiatric history. She reached the appropriate developmental milestones and, at age 1, began walking and talking. At age 11, her health began to deteriorate. Her motor movements slowed, and she had trouble sitting and standing. She also struggled with grasping objects, often dropping them. Her family also noticed abnormalities in her facial movement. Her speech progressively deteriorated, and her voice began to change. At age 13, she started to experience ataxia, resulting in leg weakness that now necessitates the use of a wheelchair. She was diagnosed with bipolar I disorder in 2008, at approximately 22 years of age, following her first psychiatric hospitalization for manic symptoms. Since then, she has been admitted to psychiatric wards multiple times a year, with an average of three hospitalizations per year.

The patient also had mild memory impairment, which affected her academic performance. She remained self-sufficient and independent at home until she was 29 years old. After this, her symptoms impaired her ability to perform everyday tasks, and she was thus provided with daily assistance. No professional intellectual quotient test has been conducted.

Her family history further revealed multiple congenital disabilities within the family tree, with a cousin being diagnosed with Joubert syndrome. Her psychiatric history revealed manic relapses approximately every three months; her last hospitalization was three months ago. During the mental state examination, she appeared alert and cooperative, with good grooming, but exhibited impaired cognition due to her medical condition and a language barrier. Her mood was euphoric, consistent with her manic state. She was diagnosed with bipolar I disorder, current episode manic, with psychotic features.

She presented with slight memory impairment, slurred speech, bladder incontinence, constipation, and an occasional pins-and-needles sensation in various parts of her body lasting 5-10 minutes, with no accompanying skin rash. During the neurological examination, the patient was conscious and fully oriented to time, place, and person. She exhibited gaze-evoked nystagmus on horizontal gaze while other cranial nerves were intact. There was no facial weakness. Extraocular movements were normal, with no limitations noted. Upper- and lower-limb muscle tone was mildly hypotonic, and muscle bulk was reduced. Motor power was as follows: neck flexors and extensors 5/5; right upper limb: proximal 5/5, distal 5/5; left upper limb: proximal 4/5, distal 4/5; right lower limb: proximal 4/5, distal 4/5; left lower limb: proximal 4/5, distal 4/5. There was bilateral hyperreflexia, with both plantar responses upgoing. Bilateral finger-nose ataxia was more pronounced on the right side, and she demonstrated an ataxic gait. Sensory examination revealed intact pinprick sensation bilaterally.

Laboratory tests for vitamin levels, alpha-fetoprotein, ceruloplasmin, and vasculitis profiles were negative. Despite adhering to her regular regimen of sodium valproate, a trough level obtained on admission, drawn prior to her next scheduled dose, showed a subtherapeutic valproic acid concentration of 27.6 micrograms/liter, which may have been clinically relevant to her manic relapse. Additionally, her prolactin level was elevated at 2706 milli-international units per liter, prompting a medication adjustment. Computed tomography of the brain revealed significant evidence of cerebellar atrophy (Figure 1). These findings suggested a chronic process in the posterior fossa, likely of developmental origin (Figure 1).

**Figure 1:**
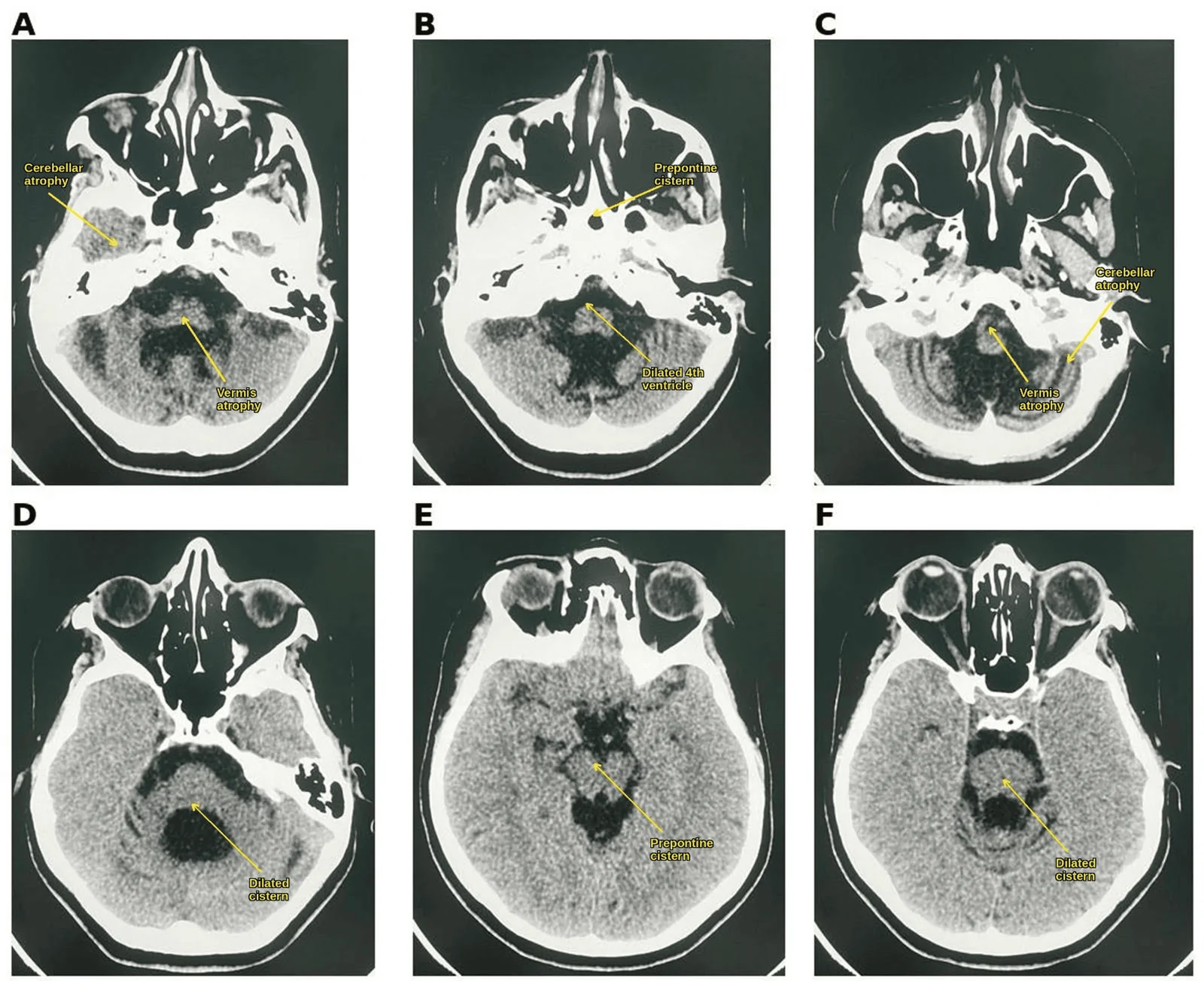
Computed tomography of the brain showing cerebellar vermian atrophy with significant dilation of the fourth ventricle and prepontine cistern (A-F) This figure represents original radiological data obtained during the patient’s diagnostic evaluation and is included with appropriate anonymization.

Given the patient's longstanding ataxia, cerebellar atrophy, and progressive motor decline, differential diagnoses for the underlying neurological presentation included other GM2 gangliosidoses (Tay-Sachs disease), other lysosomal storage disorders, hereditary spinocerebellar ataxias, Wilson's disease, and autoimmune or paraneoplastic cerebellar syndromes. Wilson's disease was considered unlikely given a normal ceruloplasmin level, and vasculitis and autoimmune screening were unremarkable. The homozygous pathogenic HEXB variant on molecular genetic testing, together with the clinical and radiological findings, confirmed Sandhoff disease as the underlying diagnosis.

The treatment plan included admission under psychiatric care with close monitoring, with a diagnosis of bipolar I disorder, current episode manic, severe, with psychotic features. Risperidone and lorazepam were initiated, while sodium valproate was up-titrated. Escitalopram was discontinued during the acute manic episode because of concern regarding potential mood destabilization.

After six days of inpatient treatment, her acute manic and psychotic symptoms improved, and she was discharged on risperidone, lorazepam, and extended-release sodium valproate (Depakene Chrono). Following discharge, depressive symptoms emerged several weeks later, characterized by low mood, reduced energy, psychomotor retardation, and social withdrawal, without psychotic or manic features. Risperidone and sodium valproate were continued concurrently at their existing doses throughout this period. Given the severity of her depressive symptoms and their impact on her functioning, escitalopram was restarted, as it had previously been an effective component of her maintenance regimen; this decision was made alongside the continuation of her mood stabilizer and antipsychotic, with the intention of closely monitoring for early signs of mood destabilization or manic switching given her history of frequent relapses. Escitalopram was subsequently discontinued at home and was not resumed by her caregiver, limiting the ability to fully assess the intended monitoring plan.

The patient then traveled to Iran for approximately six months of rehabilitation and physiotherapy while continuing her other medications. As no disease-modifying treatment exists for Sandhoff disease, management of her underlying condition was supportive and multidisciplinary, centered on rehabilitation and physiotherapy to preserve function, alongside continuation of her psychiatric medications.

At subsequent outpatient follow-up, the patient's father and sister reported that she remained psychiatrically stable on her ongoing medication regimen. No new adverse or unexpected events were reported. The family reported good tolerability of the treatment and continued participation in rehabilitation and physiotherapy.

The full clinical course, including medications, treatment response, and follow-up, is summarized in the timeline (Table 2).

**Table 2:** Timeline of clinical events across the episode of care Original data derived from the present case. WES: whole-exome sequencing

Age/relative time point	Clinical event/care milestone
Birth	Born at full term to consanguineous parents; normal early development
Age 1	Achieved independent walking and speech
Age 11	Onset of motor slowing, gait and sitting difficulty, impaired grasp, facial and speech changes
Age 13	Onset of ataxia and progressive leg weakness; eventual wheelchair dependence
2008 (age ~22)	First psychiatric hospitalization and diagnosis of bipolar I disorder with mixed features; started on escitalopram, risperidone, and sodium valproate
2008 onward	Recurrent psychiatric hospitalizations (~3/year); manic relapses roughly every 3 months
Age 29	Loss of independence in activities of daily living; began requiring daily caregiver assistance
Several years before this admission	Formal clinical diagnosis of ataxia
Pre-admission	WES identifies homozygous HEXB missense variant; genetic diagnosis of Sandhoff disease confirmed
Index admission, Day 0	Presents to emergency department with 5-day history of escalating mania, psychosis, and agitation
Index admission	Neurological examination shows cerebellar signs and hyperreflexia; CT brain shows cerebellar atrophy (Figure 1); admitted under psychiatry for bipolar I disorder, current episode manic, severe, with psychotic features
Index admission	Started on risperidone and lorazepam; sodium valproate up-titrated; escitalopram discontinued
Day 6	Discharged on risperidone, lorazepam, and sodium valproate with clinical improvement
Post-discharge	Depressive symptoms emerge; escitalopram restarted, then interrupted at home and not resumed by caregiver
Post-discharge	Travels to Iran for 6 months of rehabilitation and physiotherapy while continuing other medications
Follow-up	Returns and resumes outpatient follow-up; family reports sustained psychiatric stability on current regimen

Because of the patient's cognitive and communication difficulties, her perspective was conveyed through her father and sister, who were her primary caregivers during outpatient follow-up. They reported that, despite the initial distress associated with the acute psychiatric admission, she tolerated the medication adjustments well and preferred to continue treatment and rehabilitation in a family-supported environment. The family expressed relief regarding the psychiatric stabilization achieved and emphasized the importance of medication adherence, rehabilitation, and close follow-up in maintaining her well-being and functional independence.

## Discussion

Written informed consent for publication of this case report, including clinical and imaging details, was obtained from the patient's father as her legal guardian and primary caregiver, given her cognitive and communication difficulties.

Adult-onset Sandhoff disease presents a diagnostic challenge because neurological symptoms may develop gradually and overlap with manifestations of other neurological or psychiatric disorders. Progressive ataxia, lower-limb weakness, gait disturbance, dysarthria, and swallowing difficulties have been described in adult-onset GM2 gangliosidosis [4,5]. Neuropsychiatric manifestations may include mood disturbance, psychosis, behavioral changes, delusions, and hallucinations [4,6].

In the present case, the patient's clinical diagnosis of bipolar I disorder was established, and psychiatric hospitalizations began in late adolescence. In contrast, subtle motor symptoms had already emerged at approximately age 11, several years earlier, with progressive ataxia evident by age 13. Genetic confirmation of Sandhoff disease itself occurred only recently, well after her psychiatric diagnosis. This chronology raises the possibility that her psychiatric symptoms did not represent an entirely independent primary disorder. Instead, they may have represented an early or evolving neuropsychiatric manifestation of her underlying GM2 gangliosidosis, later compounded by progressive neurodegeneration. Distinguishing a primary psychiatric disorder from neuropsychiatric manifestations of Sandhoff disease is not possible with certainty in this case, underscoring the diagnostic complexity that arises when neurological and psychiatric symptoms evolve concurrently over time.

The patient’s progressive motor impairment began during childhood, with motor slowing and impaired grasp at approximately 11 years of age, followed by ataxia and progressive lower-limb weakness at approximately 13 years of age. These symptoms eventually resulted in wheelchair dependence and loss of functional independence. The presence of progressive cerebellar signs, hyperreflexia, gait ataxia, and cerebellar atrophy on neuroimaging supported an underlying neurological process rather than an isolated psychiatric disorder.

The diagnosis was ultimately confirmed through molecular genetic testing. The patient carried a homozygous pathogenic HEXB variant, c.1514G>A (p.Arg505Gln), consistent with autosomal recessive Sandhoff disease. Genetic confirmation is particularly important in late-onset presentations because the clinical phenotype can be heterogeneous and overlap with other neurological disorders [2,5].

The acute psychiatric episode responded to conventional psychiatric treatment with risperidone, lorazepam, and sodium valproate. However, pharmacological management of psychiatric symptoms in patients with lysosomal storage disorders requires careful consideration because underlying neurodegeneration may increase vulnerability to adverse neurological effects. Previous literature has described neuropsychiatric complications and potential treatment challenges associated with lysosomal disorders [6,8].

This case also highlights the importance of multidisciplinary management. Patients with late-onset Sandhoff disease may require coordinated input from psychiatry, neurology, genetics, rehabilitation medicine, nursing, and caregivers. Psychiatric symptoms should be assessed alongside the trajectory of neurological deterioration rather than considered in isolation.

The patient’s case further demonstrates the importance of considering an underlying metabolic or genetic disorder when a patient with psychiatric illness develops progressive neurological abnormalities. Features such as progressive ataxia, weakness, dysarthria, abnormal neurological examination findings, cognitive decline, and cerebellar atrophy should prompt appropriate neurological and metabolic investigations.

Strengths and limitations

The principal strength of this case is the combination of a longstanding psychiatric history with a genetically confirmed diagnosis of Sandhoff disease and documented progressive neurological deterioration. This provides an opportunity to illustrate the diagnostic complexity of psychiatric symptoms occurring in the context of a rare neurodegenerative disorder.

The main limitations are those inherent in a single-patient case report. The case cannot establish causality or be generalized to all patients with Sandhoff disease or bipolar disorder. As this is a single case, the temporal co-occurrence of bipolar I disorder and Sandhoff disease does not establish a causal or mechanistic relationship; whether the psychiatric illness represents an independent comorbidity or forms part of the neuropsychiatric phenotype of Sandhoff disease cannot be determined from this report alone. Some developmental and psychiatric information was obtained from family members and may therefore be subject to recall bias. Formal neuropsychological and intelligence quotient assessments were not performed. Additionally, follow-up information was incomplete during the patient’s rehabilitation period abroad.

## Conclusions

Sandhoff disease should be considered in patients with psychiatric symptoms who develop progressive neurological abnormalities, particularly ataxia, weakness, dysarthria, gait disturbance, and cerebellar signs. In patients with an established psychiatric diagnosis, new or progressive neurological findings should not automatically be attributed to the underlying psychiatric disorder. Conversely, in patients with a confirmed metabolic or neurodegenerative disorder such as Sandhoff disease, mood and psychotic symptoms should not automatically be assumed to represent an independent primary psychiatric illness; the possibility that they reflect a neuropsychiatric manifestation of the underlying disease should also be considered. Genetic and metabolic investigations can be critical for establishing the diagnosis in late-onset presentations. Management should be individualized and multidisciplinary, with careful monitoring of both psychiatric symptoms and neurological function.
